# The Patterns and Impact of Social Media Exposure of Journal Publications in Gastroenterology: Retrospective Cohort Study

**DOI:** 10.2196/25252

**Published:** 2021-05-14

**Authors:** Austin Lee Chiang, Loren Galler Rabinowitz, Javid Alakbarli, Walter W Chan

**Affiliations:** 1 Brigham and Women's Hospital Boston, MA United States; 2 Columbia University Medical Center New York, NY United States; 3 Beth Israel Deaconess Medical Center Boston, MA United States

**Keywords:** social media, gastroenterology journals, gastroenterology research, journal citations

## Abstract

**Background:**

Medical journals increasingly promote published content through social media platforms such as Twitter. However, gastroenterology journals still rank below average in social media engagement.

**Objective:**

We aimed to determine the engagement patterns of publications in gastroenterology journals on Twitter and evaluate the impact of tweets on citations.

**Methods:**

This was a retrospective cohort study comparing the 3-year citations of all full-length articles published in five major gastroenterology journals from January 1, 2012, to December 31, 2012, tweeted by official journal accounts with those that were not. Multivariate analysis using linear regression was performed to control for journal impact factor, time since publication, article type, frequency of reposting by other users (“retweets”), and media addition to tweets. Secondary analyses were performed to assess the associations between article type or subtopic and the likelihood of social media promotion/engagement.

**Results:**

A total of 1666 articles were reviewed, with 477 tweeted by the official journal account. Tweeting an article independently predicted increased citations after controlling for potential confounders (*β* coefficient=13.09; *P*=.007). There was significant association between article type and number of retweets on analysis of variance (ANOVA) (*P*<.001), with guidelines/technical reviews (mean difference 1.04, 95% CI 0.22-1.87; *P*<.001) and meta-analyses/systemic reviews (mean difference 1.03, 95% CI 0.35-1.70; *P*<.001) being retweeted more than basic science articles. The manuscript subtopics most frequently promoted included motility/functional bowel disease (odds ratio [OR] 3.84, 95% CI 1.93-7.64; *P*<.001) and education (OR 4.69, 95% CI 1.62-13.58; *P*=.004), while basic science papers were less likely tweeted (OR 0.154, 95% CI 0.07-0.34; *P*<.001).

**Conclusions:**

Tweeting of gastroenterology journal articles independently predicted higher 3-year citations. Wider adoption of social media to increase reach and measure uptake of published research should be considered.

## Introduction

Social media is playing an increasingly important role in health care as an inexpensive way to improve accessibility of medical information. In recent years, medical journals have created accounts on social media to share published content and improve visibility among both mainstream audiences and health professionals. Unlike traditional media, social media differs in its ability to curate content (using tools like hashtags) and to facilitate engagement by readers and viewers. It is often challenging to stay abreast of all newly published data from the multitude of scientific journals within a given medical specialty. Social media promotion may help narrow the range of what is considered most relevant to target audiences and/or newsworthy.

Twitter is the prime social media platform for online discussion with 335 million active monthly users worldwide and more than 500 million “tweets” per day [1]. The content of each tweet is restricted to 280 characters and may contain links to external websites. Each tweet is visible to followers of the account, and individuals can engage by reposting (ie, “retweeting”) content in their own Twitter feeds or “liking” posts.

Twitter activity may predict publication and overall journal performance. A prior study found a significant association between Twitter followers of an official journal account and both the journal impact factor and total citations [2], with an estimated 1% increase in journal citations for every 0.62% increase in Twitter followers. Among adult and pediatric urology journals, having an official Twitter account was found to be associated with a greater impact factor [3,4]. Following a targeted effort to promote published articles to their 700 followers, the *Journal of Neurointerventional Surgery* saw a “substantial increase” of 1500 visits to its scientific content [5].

The effects of social media exposure of published research in gastroenterology remain unclear. More specifically, the impacts on citations and patterns of social media promotion of these publications are also unknown. While the process that journals employ to select and promote articles on social media is largely opaque and likely not random, we may be able to glean the patterns of social media amplification and control for confounders like manuscript type and subject matter. Characterizing these patterns may serve to highlight areas that are lacking exposure or reflect what journals perceive as most relevant to the general public.

The primary aim of this study was to determine if social media promotion by gastroenterology journal Twitter accounts is associated with greater number of citations. We also aimed to determine if there is preferential promotion of certain types of publications and subtopics by gastroenterology journals on Twitter.

## Methods

This was a retrospective cohort study assessing the citations of all full-length articles published in five major peer-reviewed gastroenterology journals (*American Journal of Gastroenterology*, *Clinical Gastroenterology and Hepatology*, *Gastroenterology*, *Gastrointestinal Endoscopy*, and *Pancreas*) from January 1, 2012, to December 31, 2012. These journals and this date range were selected for the following reasons: to allow sufficient time for citations to accrue, to account for the diminishing impact of a tweet over time, and to select a year where all five journals appeared on social media.

The number of citations in academic literature as of November 15, 2015, according to Google Scholar was compared between articles tweeted by the official journal accounts and those that were not. Google Scholar has been previously used in similar research to quantify citations [6]. Publications were further categorized by manuscript type and subtopic. Manuscript types included prospective research, retrospective research, basic science, meta-analyses and systematic reviews, guidelines and technical reviews, case reports, video publications, and editorials. Manuscript subtopics included esophagus, gastric, small bowel, colon, liver, pancreas, biliary, motility and functional bowel disease, cancer, basic science, quality improvement, cost-effectiveness, inflammatory bowel disease, endoscopy, education, and microbiome. Multiple assignments for subtopics were permitted.

The mean number of citations between tweeted articles and those that were not tweeted by the official journal accounts were compared using Student *t* test. To detect an independent association between social media promotion on Twitter and Google Scholar citations, multivariate analysis using linear regression was performed to control for the 2012 journal impact factor (as published by Thomson Reuters), time since publication, and article type. Among articles that were tweeted, the overall associations between manuscript types and number of citations were assessed using analysis of variance (ANOVA). Pairwise comparisons of manuscript types with regard to number of citations were performed using the Bonferroni method.

To evaluate the likelihood of specific subtopics being promoted on social media by gastroenterology journals, the rates of tweeted manuscripts for each subtopic were compared to those not containing the corresponding subtopic using the chi-squared test. Multivariate analysis was performed using logistic regression, controlling for potential confounders including the specific journal, manuscript type, and number of citations. Results of regression analyses are expressed as raw coefficients to demonstrate the effect of each variable on citations and to better generalize these results to other settings or populations.

All statistical analyses were performed using SAS Version 9.4 (SAS Institute).

## Results

**Figure 1:**
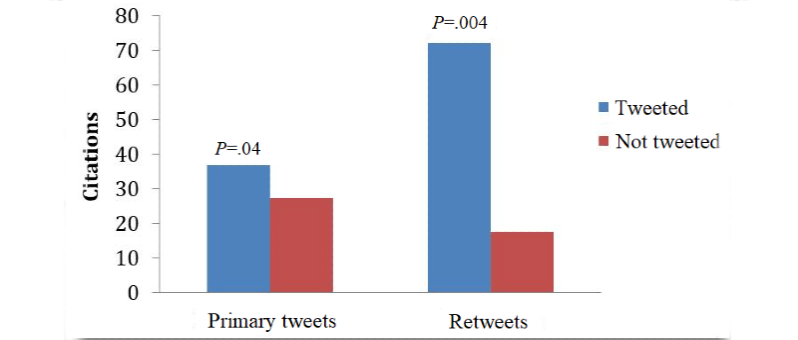
Comparison of citations between articles that were tweeted and those that were not tweeted. Primary tweets: analysis of all manuscripts (n=1666) comparing tweeted articles and nontweeted articles; retweets: analysis of all tweeted manuscripts (n=477) comparing articles that were retweeted at least once and articles without retweets.

In total, 1666 gastroenterology articles were reviewed, with 477 having been tweeted by official journal accounts. In 2012, *Gastrointestinal Endoscopy* published 451 articles, *Pancreas* published 242 articles, *American Journal of Gastroenterology* published 226 articles, *Gastroenterology* published 473 articles, and *Clinical Gastroenterology and Hepatology* published 274 articles (Table 1). On univariate analysis, articles that were tweeted had a significantly higher number of citations compared with nontweeted articles (36.9 vs 27.4, *P*=.04) (Figure 1). On multivariate analysis, tweeting of an article (*β* coefficient=13.09, *P*=.007) was independently associated with increased citations after controlling for potential confounders (Table 2). Not surprisingly, the duration since publication (in days) was found to be a predictor for increased citations in the linear regression model. Among tweeted articles, those that were retweeted (a possible proxy for strong public interest or colleague endorsement) also had higher citations compared with those that were not retweeted (72.3 vs 17.6, *P*=.004) (Figure 1), although this did not reach statistical significance in the multivariate model (*β* coefficient=23.2, *P*=.28), likely due to a small sample size.

**Table 1:** Characteristics of the articles in the five gastroenterology journals (Twitter promotion, impact factor, total citations, and frequency of publication by manuscript type and subtopic).

Characteristic		Journal						Total (N=1424)
		*AJG*^a^ (N=226)	*CGH*^b^ (N=274)	*Gastro*^c^ (N=473)	*GIE*^d^ (N=451)	*Pancreas* (N=242)		
Tweeted, n		31	69	70	137	170	477	
Not tweeted, n		195	205	403	314	72	1189	
2012 impact factor		7.282	5.627	11.675	4.878	2.386	N/A^e^	
Total citations, n		11,286	6008	21,264	8336	3201	50,095	
**Manuscript type, n**								
	Prospective studies	77	38	50	118	59	342	
	Retrospective studies	54	86	35	102	42	319	
	Basic science studies	10	1	159	6	106	282	
	Meta-analyses, systematic reviews	39	26	37	23	14	139	
	Guidelines, technical reviews	7	18	4	25	2	56	
	Editorials	34	38	58	42	5	177	
	Case reports	1	67	129	122	14	333	
	Videos	4	0	1	13	0	18	
**Publication subtopic, n**								
	Pediatric	6	4	8	9	2	29	
	Esophagus	29	38	33	55	0	155	
	Gastric	14	12	33	44	1	104	
	Small bowel	22	24	27	35	0	108	
	Colon	53	47	65	94	0	259	
	Liver	26	75	151	10	2	264	
	Pancreas	10	28	40	59	234	371	
	Biliary	8	19	19	47	1	94	
	Motility/functional	35	9	8	5	0	57	
	Cancer	22	43	89	85	124	363	
	Basis science	11	3	189	3	109	315	
	Quality improvement	19	24	21	47	4	115	
	Cost-effectiveness	1	6	2	0	1	10	
	Inflammatory bowel disease	30	26	32	4	0	92	
	Endoscopy	35	59	27	362	14	497	
	Education	2	1	9	7	0	19	
	Infectious disease/microbiome	16	7	16	3	0	42	

^a^AJG: American Journal of Gastroenterology.

^b^CGH: Clinical Gastroenterology and Hepatology.

^c^Gastro: Gastroenterology.

^d^GIE: Gastrointestinal Endoscopy.

^e^N/A: not applicable.

**Table 2:** Multivariate linear regression analysis assessing the predictors of the number of Google Scholar citations among all articles published by five major gastroenterology journals in 2012 (N=1666, tweeted n=477).

Variable		*β* coefficient (SE)	*P* value
Tweeted article		13.09 (4.82)	.007
Time since publication (days)		0.06 (0.02)	.003
**Journal**			
	*GIE^a^*	Reference	Reference
	*Gastro^b^*	37.8 (5.65)	<.001
	*AJG^c^*	23.8 (6.71)	<.001
	*CGH^d^*	3.18 (6.12)	.60
	*Pancreas*	−12.2 (7.12)	.09
**Manuscript type**			
	Prospective studies	Reference	Reference
	Retrospective studies	−8.88 (6.20)	.15
	Basic science studies	−23.5 (7.02)	<.001
	Meta-analyses/systematic reviews	26.0 (7.98)	.001
	Guidelines, technical reviews	52.8 (11.5)	<.001
	Editorials	−41.9 (7.42)	<.001
	Case reports	−43.2 (6.40)	<.001
	Videos	−21.8 (19.2)	.26

^a^GIE: Gastrointestinal Endoscopy.

^b^Gastro: Gastroenterology.

^c^AJG: American Journal of Gastroenterology.

^d^CGH: Clinical Gastroenterology and Hepatology.

On univariate analysis, articles identified in the categories of pancreas (odds ratio [OR] 3.80, *P*<.001), cancer (OR 2.20, *P*<.001), and quality improvement (OR 2.05, *P*=.003) were associated with increased Twitter promotion, while small bowel (OR 0.62, *P*=.05) and basic science (OR 0.63, *P*=.002) articles were associated with less social media exposure.

**Figure 2:**
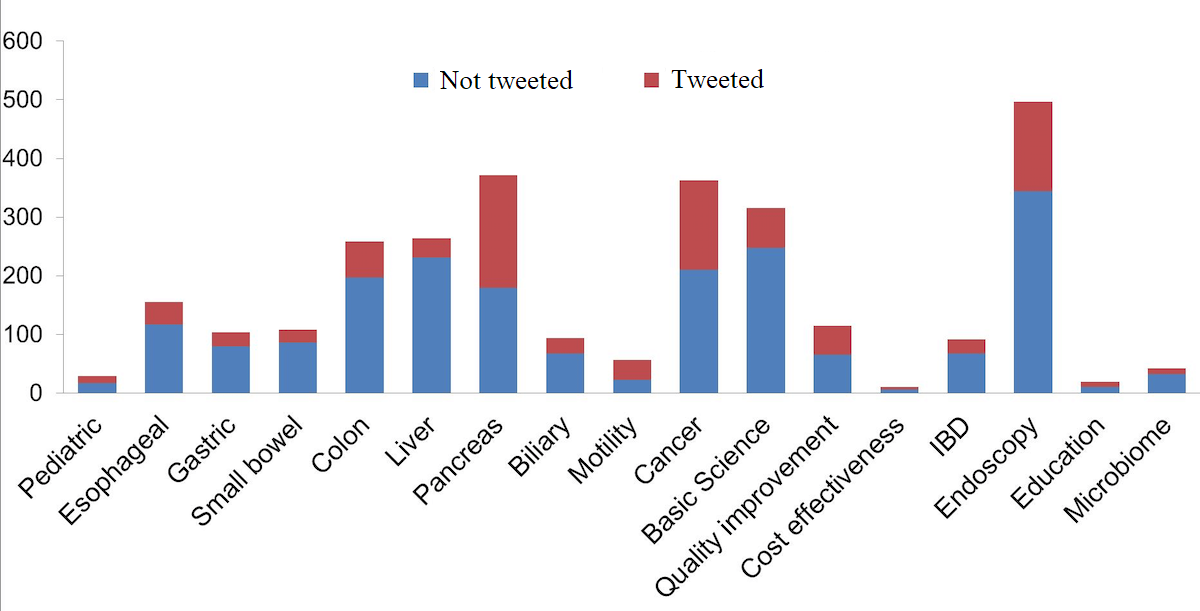
Number of publications (both tweeted and nontweeted) in 2012 by subtopic among the five journals included. IBD: inflammatory bowel disease.

After controlling for other covariates, including other manuscript subtypes, on multivariate analysis, the subtopics of motility and functional bowel disease (OR 3.84, 95% CI 1.93-7.64, *P*<.001), cancer (OR 1.392, 95% CI 1.016-1.909, *P*=.04), education (OR 4.69, 95% CI 1.62-13.58, *P*=.004), and quality improvement (OR 2.41, 95% CI 1.52-3.84, *P*<.001) were independently associated with increased promotion on Twitter, while basic science articles were significantly less likely to be tweeted (OR 0.154, 95% CI 0.07-0.34, *P*<.001) (Figure 2).

**Figure 3:**
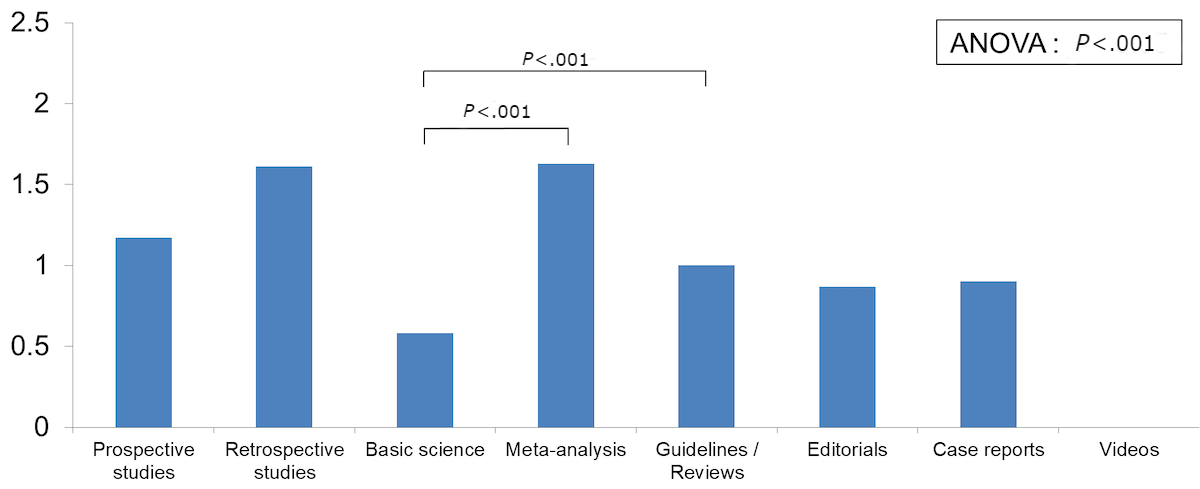
Mean number of retweets by article type.

A significant overall association between article type and citations was noted on ANOVA (*P*<.001), with guidelines/technical reviews (mean 90.23, SD 355.57), meta-analyses/systemic reviews (mean 75.22, SD 124.85), and prospective studies (mean 42.96, SD 54.26) having the most citations. On multivariate analysis using prospective studies as a reference, guidelines/technical reviews (*β* coefficient=52.8, *P*<.001) and meta-analyses/systemic reviews (*β* coefficient=26, *P*=.001) were significantly more likely to be cited, while basic science articles (*β* coefficient=−23.5, *P*<.001), case reports (*β* coefficient=−43.2, *P*<.001), and editorials (*β* coefficient=−41.9, *P*<.001) had significantly fewer citations (Table 2). Among tweeted articles, there was a significant association between article type and number of retweets on ANOVA (*P*<.001). On pairwise comparison, guidelines/technical reviews (mean difference 1.04, 95% CI 0.22-1.87, *P*<.001) and meta-analyses/systemic reviews (mean difference 1.04, 95% CI 0.22-1.86, *P*<.001) were being retweeted significantly more than basic science articles (Figure 3). Media additions to tweets were not associated with the number of citations or retweets.

## Discussion

### Principal Findings

Our study showed that social media promotion of gastroenterology journal publications on Twitter independently predicted a greater number of Google Scholar citations in 3 years after controlling for journal impact factor, type of article, and time since publication.

We also found that tweeted articles that were retweeted had significantly increased citations, perhaps a reflection that public engagement with these tweets might predict further academic interest. Our study also demonstrated that guidelines/technical reviews and meta-analyses/systematic reviews are among the most heavily promoted article types by official journal accounts on Twitter. Articles involving motility/neurogastroenterology, cancer, education, and quality improvement were independently associated with increased social media exposure, while basic science manuscripts were less promoted. This may reflect the perceived areas of public interest in gastroenterology and influence perception of gastroenterology research, resulting in potential secondary impacts on factors, such as funding. Further studies are needed to evaluate why these promotional differences exist and how this might affect research funding availability, public interest, and clinical and epidemiological outcomes.

As done in studies from other disciplines, Twitter was chosen given that it is the predominant mainstream social media platform where journal publications are regularly promoted, where academic discussion exists, and where analytics are most readily available. The evidence regarding the association between Twitter promotion and publication citations has varied across different disciplines. One study of 20 ecological journals showed a similar positive correlation between social media exposure and citations, independent of time since publication and impact factor [7]. In the *American Journal of Psychiatry*, a study of 438 published articles suggested that a greater frequency of Twitter mentions was associated with more citations [8]. One study of 286 articles from the *Journal of Medical Internet Research* showed that highly tweeted articles were 11 times more likely to be highly cited and that Twitter mentions in the first 3 days could predict highly cited articles [6].

However, this observation was not consistent across all disciplines. In a randomized trial of social media impact on 243 publications from *Circulation*, there was no significant association between social media exposure and 30-day article website views according to Google analytic data [9]. There were also no differences noted by article subtype (referring to the general categories of clinical, population, or basic science articles). One study of all 1.4 million biomedical publications between 2010 and 2012 found a weak correlation between Twitter mentions of journal articles and traditional bibliometric indicators, although the authors concluded that Twitter mentions did not reflect traditional research impact [10].

The number of annual citations by a journal is used to calculate a journal’s impact factor [11]. Historically, journal impact factor has been directly correlated with citations, as impact factor is the ratio of the number of citations to the number of publications by a journal in a given year. Google Scholar was chosen as the source of citations given its inclusivity of citations among all sources, unlike Web of Science and Scopus, which only consider citations from within journals listed in their libraries. However, some researchers have raised concerns that certain tactics can be used to boost the journal impact factor, such as publishing more review articles, strategic publication timing, and internal citations. Citations also take time to accumulate. Social media may therefore play another role in measuring the societal impact of an individual article. As a result, other metrics collectively known as “altmetrics,” which include not only number of citations, but also social media views and engagement, references by databases, and news media, are emerging [12].

In our study, guidelines and reviews published in major gastroenterology journals were the most heavily promoted types of manuscripts on social media. This promotional pattern may be a result of known interest in these articles, given existing knowledge that guidelines and reviews accrue more citations than other publication types, and they may be more relevant to a broader audience that includes general clinicians and practitioners [13]. For gastrointestinal subtopics, our finding of functional bowel disease and cancer articles as the most tweeted is also not surprising. Communities for these conditions are very active on social media, and journals may be preferentially promoting these articles knowing the discussion and engagement they tend to generate. Moreover, the high general prevalence of functional bowel conditions and the increased interest cancer-related topics tend to generate in the lay media may also play roles in the higher social media activities of these articles.

Social media engagement by the gastroenterology community is lagging behind other medical subspecialties. Adoption of Twitter by journals still varies widely by specialty, with 70% of the 20 leading radiology journals on Twitter, but only 28.1% of general medical journals and 39% of urological journals on Twitter [2,14]. Latest data suggest that the proportion of overall tweeted gastroenterology publications (7.8%) falls below the average rate of Twitter promotion (9.4%) of all medical journal articles [10]. A survey of 265 gastroenterologists in 2015 found that 82.1% of respondents did not access social media for journals or other educational purposes, and 47.7% reported never having used any form of social media. This is in contrast to patient acceptance of social media, where 84.4% (n=112) of inflammatory bowel disease patients and 72.9% (n=68) of chronic viral hepatitis patients favored interaction with health care professionals on social media [15]. This dynamic may be improving in recent years, as there has been a growing presence of gastroenterologists and young physicians on social media.

One limitation of this study is the inability to establish causality and elucidate the potential mechanisms of social media impact on citations. Articles addressing subjects of higher interest or “more popular” topics (therefore, more highly cited) may be selectively tweeted at a higher frequency. For instance, social media promotion has been associated with greater downloads of journal articles in clinical pain sciences, which could suggest greater readership as a catalyst for future citations [16]. The radiology community has observed a greater distribution of web links to published articles on Twitter [14]. In fact, the effect of social media on journal article readership has been previously demonstrated in a study of the *Journal of the American College of Radiology*, which showed that a planned Twitter-based discussion increased monthly website journal article views by 31.4%, unique visitors by 20.0%, and website visits by 25.5% [17]. In a randomized controlled study of the same journal, Twitter promotion of publications was independently associated with significantly greater weekly webpage visits (18.2 vs 7.6 page visits) [17]. Another study showed that 7 out of 11 radiology journals new to Twitter experienced increases in impact factor after 1 year [14].

Moreover, it is unknown if social media alone can be credited for greater citations or if social media is simply one arm of a larger promotional effort that includes other avenues, such as press releases for traditional media coverage. It is worth noting, however, that because the initial promotional tweet precedes article downloads and subsequent citations, it is not possible for the number of citations to cause a reactionary increase in social media presence for that particular article. Additionally, various journals may adopt different methods to selectively promote certain subtopics or manuscript types (such as guidelines) on social media. To best account for these biases, we controlled for subtopics and manuscript types in the multivariate analysis. Another potential limitation was the potential overestimation of baseline academic impact using Google Scholar citations. Though Google Scholar may include duplicate citations or citations of a paper in a nonpeer-reviewed publication without scholarly relevance, the Google algorithm is standard and therefore objectively compares citation volume.

### Conclusion

In conclusion, social media promotion on Twitter of gastroenterology publications independently predicted a greater number of citations in 3 years in this exploratory study. Publications and researchers should consider wider adoption of social media to increase reach and measure uptake of published research. Social media promotion of publications can not only potentially boost journal citations, but also help define the societal impact of an individual article and thus influence academic promotion. However, beyond citations and academic uptake, journals and physicians should be aware of other benefits of social media for professionals, patients, and family members. In addition to academic productivity, it is important to recognize how social media could have other important roles in public health. For instance, social media may help propel academic medicine by informing other professionals. Moreover, it is our public health responsibility as a medical community to serve as primary sources of accurate up-to-date medical information online in order to preserve the integrity of what readers consume. Internally, social media can provide an open forum for discussion, boost professional and institutional recognition, attract referrals for trial enrollment and research purposes, and perhaps encourage funding to sustain academic research.

## Abbreviations

ANOVA: analysis of variance
OR: odds ratio

## References

[1] Q2 2018 Selected Company Financials and Metrics. Twitter.

[2] Cosco TD (2015). Medical journals, impact and social media: an ecological study of the Twittersphere. CMAJ.

[3] O'Kelly F, Nason G, Manecksha R, Cascio S, Quinn F, Leonard M, Koyle M, Farhat W, Leveridge M (2017). The effect of social media (#SoMe) on journal impact factor and parental awareness in paediatric urology. J Pediatr Urol.

[4] Nason GJ, O'Kelly F, Kelly ME, Phelan N, Manecksha RP, Lawrentschuk N, Murphy DG (2015). The emerging use of Twitter by urological journals. BJU Int.

[5] Fargen KM, Ducruet AF, Hyer M, Hirsch JA, Tarr RW (2017). Expanding the social media presence of the Journal of Neurointerventional Surgery: editor's report. J Neurointerv Surg.

[6] Eysenbach G (2011). Can tweets predict citations? Metrics of social impact based on Twitter and correlation with traditional metrics of scientific impact. J Med Internet Res.

[7] Peoples BK, Midway SR, Sackett D, Lynch A, Cooney PB (2016). Twitter Predicts Citation Rates of Ecological Research. PLoS One.

[8] Quintana DS, Doan NT (2016). Twitter Article Mentions and Citations: An Exploratory Analysis of Publications in the American Journal of Psychiatry. Am J Psychiatry.

[9] Fox CS, Bonaca MA, Ryan JJ, Massaro JM, Barry K, Loscalzo J (2015). A randomized trial of social media from Circulation. Circulation.

[10] Haustein S, Peters I, Sugimoto CR, Thelwall M, Larivière V (2013). Tweeting biomedicine: An analysis of tweets and citations in the biomedical literature. J Assn Inf Sci Tec.

[11] Garfield E (2006). The history and meaning of the journal impact factor. JAMA.

[12] Scarlat MM, Mavrogenis AF, Pećina M, Niculescu M (2015). Impact and alternative metrics for medical publishing: our experience with International Orthopaedics. International Orthopaedics (SICOT).

[13] Haustein S, Costas R, Larivière V (2015). Characterizing social media metrics of scholarly papers: the effect of document properties and collaboration patterns. PLoS One.

[14] Kelly BS, Redmond CE, Nason GJ, Healy GM, Horgan NA, Heffernan EJ (2016). The Use of Twitter by Radiology Journals: An Analysis of Twitter Activity and Impact Factor. J Am Coll Radiol.

[15] Timms C, Forton D, Poullis A (2014). Social media use in patients with inflammatory bowel disease and chronic viral hepatitis. Clin Med (Lond).

[16] Allen HG, Stanton TR, Di Pietro F, Moseley GL (2013). Social media release increases dissemination of original articles in the clinical pain sciences. PLoS One.

[17] Hawkins CM, Hillman BJ, Carlos RC, Rawson JV, Haines R, Duszak R (2014). The impact of social media on readership of a peer-reviewed medical journal. J Am Coll Radiol.

